# Differential Involvement of *Arabidopsis* β’-COP Isoforms in Plant Development

**DOI:** 10.3390/cells11060938

**Published:** 2022-03-09

**Authors:** Judit Sánchez-Simarro, Pilar Selvi, César Bernat-Silvestre, Eugenio Gómez Minguet, Fernando Aniento, María Jesús Marcote

**Affiliations:** 1 Instituto Universitario de Biotecnología i Biomedicina (BIOTECMED), Departamento de Bioquímica y Biología Molecular, Facultad de Farmacia, Universitat de València, 46100 Burjassot, Spain; judit.sanchez-simarro@uv.es (J.S.-S.); pilar.selvi@uv.es (P.S.); cesar.bernat@uv.es (C.B.-S.); 2 Instituto de Biología Molecular y Celular de Plantas (CSIC-UPV), Ciudad Politécnica de la Innovación, Edif. 8E. C/Ingeniero Fausto Elio s/n, 46011 Valencia, Spain; egomezm@ibmcp.upv.es

**Keywords:** *Arabidopsis*, plant growth, coat protein I (COPI), isoforms, β’-COP, α-COP

## Abstract

Coat protein I (COPI) is necessary for intra-Golgi transport and retrograde transport from the Golgi apparatus back to the endoplasmic reticulum. The key component of the COPI coat is the coatomer complex, which is composed of seven subunits (α/β/β’/γ/δ/ε/ζ) and is recruited *en bloc* from the cytosol onto Golgi membranes. In mammals and yeast, α- and β’-COP WD40 domains mediate cargo-selective interactions with dilysine motifs present in canonical cargoes of COPI vesicles. In contrast to mammals and yeast, three isoforms of β’-COP (β’1-3-COP) have been identified in *Arabidopsis*. To understand the role of *Arabidopsis* β’-COP isoforms in plant biology, we have identified and characterized loss-of-function mutants of the three isoforms, and double mutants were also generated. We have found that the trafficking of a canonical dilysine cargo (the p24 family protein p24δ5) is affected in *β’-COP* double mutants. By western blot analysis, it is also shown that protein levels of α-COP are reduced in the *β’-COP* double mutants. Although none of the single mutants showed an obvious growth defect, double mutants showed different growth phenotypes. The double mutant analysis suggests that, under standard growth conditions, *β’1-COP* can compensate for the loss of both *β’2-COP* and *β’3-COP* and may have a prominent role during seedling development.

## 1. Introduction

Coat Protein I (COPI)-coated vesicles are involved in transport between Golgi cisternae and in retrograde transport from the Golgi apparatus back to the endoplasmic reticulum (ER) [1]. In mammalian cells, COPI proteins have been recently proposed as also playing a role in the last step of anterograde ER–Golgi transport [2,3].

The COPI coat is based on a cytosolic complex (coatomer), containing seven equimolar subunits (α–, β–, β’–, γ–, δ–, ε– and ζ-COP), which interacts with Golgi membranes via the GTPase ADP-ribosylation factor 1 (ARF1). Cytosolic (GDP-bound) ARF1 first interacts with dimers of p24 family proteins, but following GTP/GDP exchange, ARF1–GTP dissociates from p24 proteins and inserts into Golgi membranes. Coatomer can then interact both with ARF1–GTP and with sorting signals (i.e., dilysine motifs) in the cytosolic domain of p24 family proteins and other COPI cargo proteins. Coatomer polymerization induces COPI vesicle formation, whereas COPI uncoating requires GTP hydrolysis in ARF1 [1,4,5]. In contrast to clathrin and COPII coats, where the inner layer (involved in cargo recognition) and the outer layer (involved in membrane deformation) are recruited sequentially, COPI coatomer is recruited *en bloc* from the cytosol onto Golgi membranes [5,6]. However, biochemical studies have shown that coatomer is composed of two subcomplexes, the B-subcomplex, containing the α-, β’- and ε-COP subunits, and the F-subcomplex, containing the β-, γ-, δ- and ζ-COP subunits. The F-subcomplex is structurally very similar to clathrin adaptors and has been shown to interact with two molecules of ARF1–GTP. Therefore, this subcomplex has been proposed to function as an adaptor-like subcomplex. The B-subcomplex has a domain organization very similar to that of clathrin, including two N-terminal β-propeller domains followed by an α-solenoid domain [7], and indeed the crystal structure of “trimeric” α/β’-COP strikingly resembles that of a clathrin triskelion [8]. Therefore, the B-subcomplex has been proposed to function as a cage-like subcomplex. Despite this structural similarity, clathrin and the COPI B-subcomplex share no sequence similarity and evolved independently [9]. In the B-subcomplex, α- and β’-COP can be considered structural homologs, since both contain two β-propeller domains and a α-solenoid domain and can interact via the α-solenoid region to form a heterodimer [8].

Cargo recognition by the COPI complex is mediated by sorting signals on the cytosolic domain of transmembrane proteins. The best characterized sorting signals are the dilysine KKxx and KxKxx motifs, present in many ER-resident membrane proteins, including proteins of the p24 family [4,6,10,11,12,13,14,15,16]. These motifs directly bind to the N-terminal β-propeller domains (containing WD-40 repeats) of α- or β’-COP, that are ideally positioned adjacent to the surface of the membrane [6,11,12,13]. The FFxxBB(x)n motif (in which B is a basic amino acid) that is present in the cytosolic tail of several p24 family proteins binds two distinct sites in the γ-COP subunit [17]. Dilysine motifs also seem to be responsible for sorting of the K/HDEL receptor (involved in retrograde Golgi–ER transport of luminal ER-resident proteins) within COPI vesicles [18]. Other COPI sorting signals include Arginine(R)-based ER retrieval signals (φRxR, in which φ represents a hydrophobic amino acid), which interact with the beta and delta subunits [19]. Segments of six of the seven COPI subunits are membrane proximal, which makes it possible that different COPI subunits are involved in cargo recognition [6].

In yeast, all COPI subunits are encoded by single genes. Six of the seven subunits are essential for viability in *Saccharomyces cerevisiae*. Only ε-COP, emerging last during evolution, is non-essential [20]. The COPI pathway is essential for life, and depletion of COPI subunits is lethal in mammalian cells [21,22]. In mammals, two coatomer subunits (γ- and ζ-COP) have two paralogs (γ1/γ2, ζ1/ζ2), which has led to the proposal that they may be part of different COPI vesicles [4]. However, a proteomic analysis of paralog-specific COPI vesicles generated *in vitro* from HeLa cells did not show any significant difference in their cargo content, suggesting that these paralogs may be functionally redundant [23]. Nevertheless, the COPI pathway may also have tissue-/cell-type-specific functions. Indeed, mutations in COPI subunits have been linked to diseases, in particular, neurological disorders [24] and, very recently, COPI vesicles have been proposed to have a paralog-specific role in the neuronal differentiation of mouse pluripotent cells [25].

*Arabidopsis* (and most plant species) possesses several isoforms for most COPI subunits. In *Arabidopsis*, all COPI subunits (except δ- and γ-COP) have two to three isoforms, including α- (α1 and α2) and β’ (β’1, β’2 and β’3)-COP, with a potential role in cargo binding (i.e., proteins with a dilysine motif) [14]. Therefore, it is tempting to postulate that different COPI paralogs may be part of different types of COPI vesicles. Interestingly, in contrast to mammals, morphologically different COPI vesicles have been detected in plants [26]. However, it is not yet known whether the different isoforms of each COPI subunit are functionally redundant, or else may form different populations of COPI vesicles, perhaps containing different cargo proteins.

Silencing of *β’-*, *γ-* and *δ-COP* caused growth arrest and acute plant cell death in *Nicotiana benthamiana*, while silencing of *β’-COP* in tobacco BY-2 cells caused aberrant cell plate formation during cytokinesis [27]. Knockdown of ε-COP subunit isoforms in *Arabidopsis* changed the localization of endomembrane proteins, containing a KXD/E motif involved in COPI binding and Golgi localization [28]. *Arabidopsis* α1-COP has been shown to be required for the early acceptance of compatible pollen grains [29] and α2-COP for early secretory traffic and plant growth [30]. Besides, mutants in plant COPI subunits have been shown to have an altered Golgi morphology, which highlights the importance of COPI function for Golgi structure [27,28,30,31]. Loss of COPI function has also been shown to affect tolerance to salt stress, in particular to chloride ions, possibly due to mislocalization or reduced activity of chloride channels/transporters [32,33]. Here we have used a loss-of-function approach to analyze the possible function of the three paralogs of the β’-COP subunit in *Arabidopsis*.

## 2. Materials and Methods

### 2.1. Plant Material and Stress Treatments

*Arabidopsis thaliana* (ecotype Col-0) was used as wild type. The loss-of-function mutants *β’1-cop-1* (SALK_206753), *β’1-cop-2* (WiscDsLoxHs036_02G), *β’2-cop-1* (SALK_056771), *β’3-cop-1* (SALK_004817), *β’3-cop-2* (SALK_206870) and *β’3-cop-3* (SALK_096549) were from the Salk Institute Genomic Analysis Laboratory and obtained from the Nottingham *Arabidopsis* Stock Centre. In *β’1-cop-1* and *β’3-cop-2* mutants, next-generation sequencing detected only one T-DNA insertion. Mutant lines were characterized by PCR (Supplementary Table S1)*. A. thaliana* plants were grown in growth chambers under a 16-h-light:8-h-dark regime and 75% relative humidity at 21 °C. To study whether salt tolerance was affected in the *β’-COP* double mutants, seeds of wild type (Col-0) and mutants were sown on Murashige and Skoog (MS) plates containing 135–150 mM NaCl. Plates were transferred to a controlled growth chamber after cold treatment in the dark for three days at 4 °C. After 12 days, the rates of cotyledon greening were scored. To study KCl (100–110 mM) tolerance, the same protocol was used. Seeds harvested from Col-0 and mutant plants grown under the same conditions and at the same time were used.

### 2.2. Reverse Transcription PCR (RT-PCR)

Total RNA was extracted from seedlings using a NucleoSpin RNA plant kit (Macherey-Nagel, Düren, Germany), and 3 μg of the RNA solution were reverse transcribed using the maxima first strand cDNA synthesis kit for quantitative RT-PCR (Fermentas, Burlington, ON, Canada), according to the manufacturer’s instructions. Semi-quantitative PCRs (sqPCRs) were performed on a cDNA template using the PCR Master kit (EmeraldAmp Max–2X Premix) (TaKaRa Bio, Shiga, Japan). The sequences of the primers used for PCR amplifications are included in Supplementary Table S2. Quantitative PCR (qPCR) was performed by using a StepOne Plus machine (Applied Biosystems, California, CA, USA) with SYBR Premix Ex Taq TM (Tli RNaseH Plus) (TaKaRa Bio), according to the manufacturer’s protocol. Each reaction was performed in triplicate with 100 ng of the first-strand cDNA and 0.3 mM of primers for all the genes and 0.9 mM for *SEC31A* in a total volume of 20 μL. The specificity of the PCR amplification was confirmed with a heat dissociation curve (from 60 to 95 °C). Relative mRNA abundance was calculated using the comparative Ct method, according to Pfaffl [34]. Primers used for qPCR are listed in Supplementary Table S3.

### 2.3. Transgenic RFP–p24δ5 Plants

Transgenic plants were generated by transformation of Col-0, *β’1β’3-cop-1* and *β’2β’3-cop-2* plants with the RFP–p24δ5 construct via *Agrobacterium* using the floral dip method, according to standard procedures [35]. The RFP–p24δ5 construct has been previously described and encodes a RFP fusion protein with a mRFP located at the N-terminus of the protein (right after the signal sequence and before the N-terminus of the mature p24δ5 protein) under the control of the 35S promoter [30,36]. The fluorescence of the mRFP used has been shown to be highly stable at the acidic pH of the vacuole lumen [36,37]. T1 plants were analyzed by confocal microscopy.

### 2.4. Confocal Microscopy

Imaging was performed using an Olympus FV1000 confocal microscope with a 60× water lens. A fluorescence signal for RFP (543 nm/593–636 nm) was detected. Sequential scanning was used to avoid any interference between fluorescence channels. Post-acquisition image processing was performed using the FV10-ASW4.2 Viewer^®^.

### 2.5. Generation and Identification of CRISPR–Cas9 Mutants

The sequences of the three *β’-COP* genes were used with ARES-GT software (https://github.com/eugomin/ARES-GT, accessed on 21 December 2021) [38] for selection of two *β’2-COP* specific sgRNA sequences targeting exons 5 and 7. The CRISPR–Cas9 constructs were designed using the GoldenBraid 3.0 assembly approach (https://gbcloning.upv.es/, accessed on 21 December 2021) [39]. The gRNAs were assembled together using the multiplexing strategy: sgRNA1 (TTCGCACTATGTGATGCAAG) and sgRNA2 (GTTGTGTCCAGACGCTAGAT) were cloned into level 0 vectors GB1208 and GB1207 and then assembled into vector pDGB3_alpha2 (GB0017) with U6-26 promoter (GB1001). Assembled sgRNAs were combined with EGM005 plasmid [40] (pDGB3_alpha1_pAt2S3::DsRED::T35S_pUBQ10::hCas9:Tnos_SF) into vector pDGB3_omega1 (GB0019) to generate the final CRISPR–Cas9 vector. This final vector was introduced into Col-0 and *β’3-cop-2* mutant plants by floral dip for *Agrobacterium*-mediated transformation [35]. An Olympus SZX9 microscope with DsRED filter was used for the selection of fluorescent T1 transformed seeds and non-fluorescent T2 Transgen-Free seeds.

To confirm CRISPR–Cas9-mediated editing of the target gen, young leaves’ genomic DNA was obtained following the protocol described by Edwards [41] and the PCRs were performed using specific primers (Supplementary Table S4). For Sanger sequencing, obtained PCR products were purified and sequenced by Macrogen Co. (Madrid, Spain). The sequencing was carried out using a specific primer (Supplementary Table S4). Chromatograms from sequencing results were analyzed by Synthego “ICE CRISPR analysis tool” (https://ice.synthego.com/#, accessed on 13 January 2022).

### 2.6. Protein Extracts, SDS-PAGE and Immunoblotting

Cotyledons of seven-day-old wild type plants and loss-of-function mutants were ground in homogenization buffer (HB, 0.3 M sucrose; 1 mM EDTA; 20 mM KCl; 20 mM HEPES pH 7.5) supplemented with 1 mM DTT and a Protease Inhibitor Cocktail (Sigma-Aldrich Co., St. Louis, MO, USA), using a mortar and pestle. The homogenate was centrifuged two times for 5 min at 1200× *g* and 4 °C, and the post nuclear supernatant (PNS) was collected. Then, the PNS was centrifuged for 10 min at 1,000,000× *g* and 4 °C, and the supernatant was collected as a cytosolic extract.

Protein quantitation was performed by using the Bradford assay (Bio-Rad Laboratories GmbH, Munich, Germany). Protein extracts were resolved by SDS polyacrylamide gel electrophoresis (SDS–PAGE) and proteins were then transferred to nitrocellulose membranes (Schleicher and Schuell, Maidstone, UK). Membranes were stained with Ponceau S solution (Sigma) before incubation with primary antibodies against COPI subunits and peroxidase-labeled secondary antibodies. The luminescent signal was developed using the SuperSignal West Pico chemiluminescent substrate (Pierce-Thermo Scientific, Rockford, IL, USA). Polyclonal antibodies against mammalian β′-COP (C1PL) and α-COP were kindly provided by Dr F. Wieland (Biochemie-Zentrum, Heidelberg, Germany). Immunoblots were analyzed using the ChemiDoc XRS + imaging system (Bio-Rad, California, CA, USA). Immunoblots in the linear range of detection were quantified using Quantity One software (Bio-Rad Laboratories), with the Ponceau stain protein as a loading control.

### 2.7. Statistical Analysis

Differences in stress responses, protein levels in western blotting analysis and mRNA levels in RT-sqPCR were tested using a two-sample *t*-test with unequal variances (Microsoft Excel 2013) among all the *β’-COP* mutants compared to Col-0.

## 3. Results

### 3.1. Arabidopsis β’-COP Genes

Three *β’-COP* genes were identified in *Arabidopsis*: *β’1-COP* (At1g52360), *β’2-COP* (At3g15980) and *β’3-COP* (At1g79990). They all encode proteins between 104–105 kDa. β’1-COP and β’2-COP share 88%, β’1-COP and β’3-COP, 85% and β’2-COP and β’3-COP, 81% amino acids, respectively (Supplementary Figure S1).

**Figure 1:**
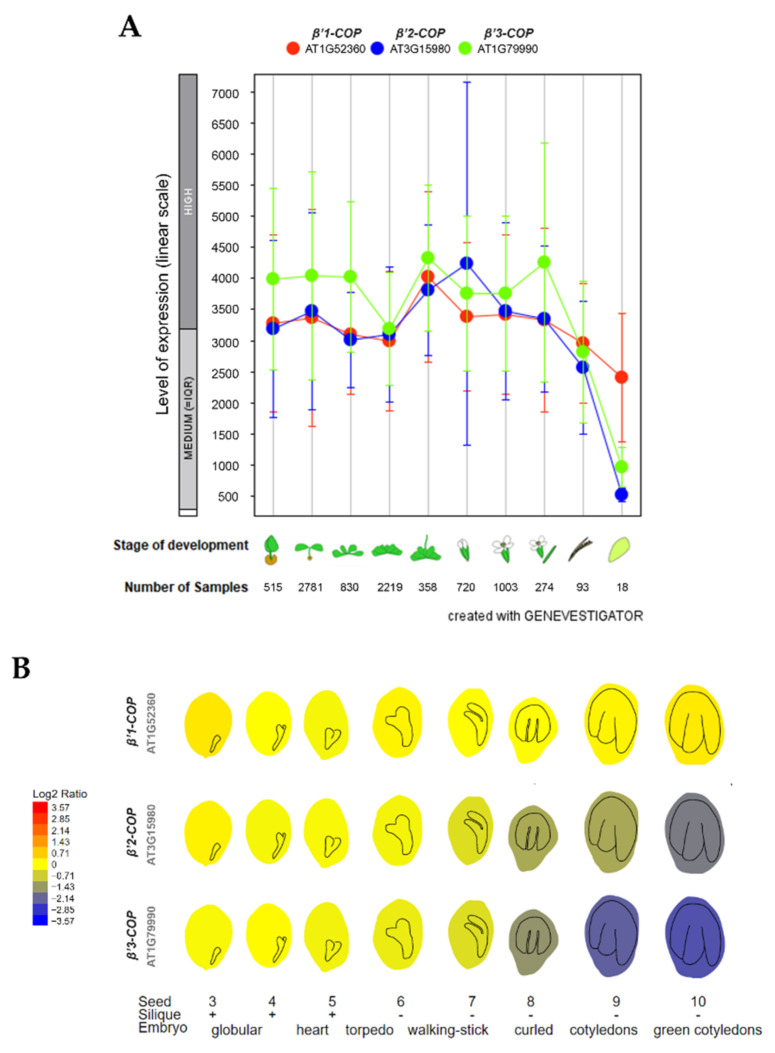
Expression patterns of *β’1-COP*, *β’2-COP* and *β’3-COP*. (**A**) Developmental stage-specific expression pattern in *Arabidopsis thaliana*. Seedlings, rosette leaves, floral organs and siliques are sequentially marked from left to right. “HIGH”, “MEDIUM” and “LOW” expressions were calculated by Afflymetrix *Arabidopsis* ATH1 genome array. The number of samples indicates RNA gene expression data collected by GENEVESTIGATOR (www.genevestigator.com, accessed on 23 November 2021). (**B**) Seed development expression pattern, from the globular embryo to the green cotyledons seed stage. *β’1-COP* shows the highest expression. Data collected and image generated by AtGenExpress eFP (http://bar.utoronto.ca/eplant, accessed on 15 December 2021) [44,45,46]. Gene expression data generated by the Affymetrix ATH1 array are normalized by the GCOS method, TGT value of 100. Tissues were sampled in triplicate. The legend at the left presents relative expression levels coded by colours (blue = low, red = high).

To investigate the relative expression of *β’-COP* genes, we used the publicly available expression database GENEVESTIGATOR [42,43] and ePlant-BAR [44]. As shown in Figure 1A, the three genes show high expression levels throughout plant development. The main difference between the three isoforms is their expression during seed development. As it is shown in Figure 1B, *β’1-COP* had the highest expression levels, up to four times higher than *β’2-COP* and six times higher than *β’3-COP*, in the last stages of seed development. This suggests that *β’1-COP* might exert a function in seed development. In contrast, *β’3-COP* showed the lowest expression levels at these stages.

**Figure 2:**
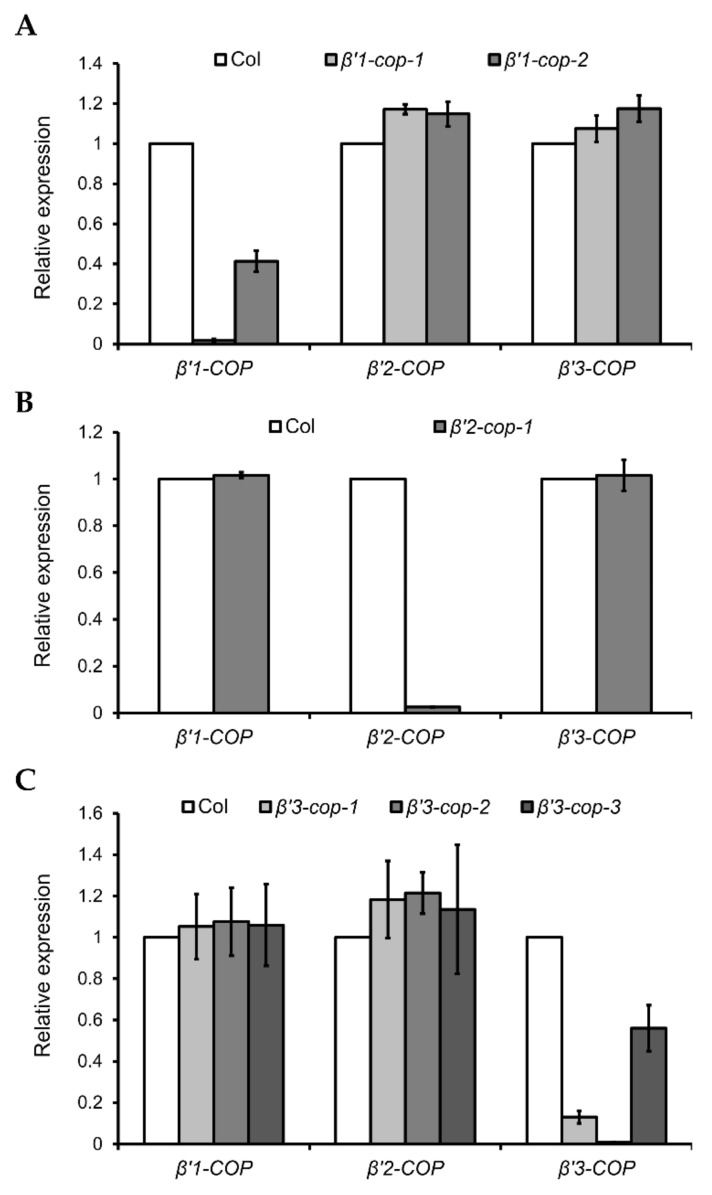
Relative expression levels of *β’-COP* genes in *β’-cop* mutants. RT–qPCR analysis was performed to characterize *β’1-cop* (**A**), *β’2-cop* (**B**) and *β’3-cop* (**C**) mutants. Total RNA was extracted from 7-day-old seedlings of the mutants and wild type (Col-0). The mRNA was analyzed by RT–qPCR with specific primers and normalized to *UBQ10* expression (Supplementary Tables S1–S3). Results are from three biological samples and three technical replicates. mRNA levels are expressed as relative expression levels and represent fold changes of mutant/wild type. Values represent mean ± s.e.m. of the three biological samples.

To gain insight into the physiological role of the three *Arabidopsis β’-COP* genes, T-DNA mutants were analyzed. Two *β’1-cop* T-DNA insertion mutants from the SALK collection (http://signal.salk.edu/cgi-bin/tdnaexpress, accessed on 7 March 2022), *β’1-cop-1* (SALK_206753) and *β’1-cop-2* (WiscDsLoxHs036_02G), were characterized (Figure 2A and Figure S2). No *β’1-COP* mRNA could be detected in *β’1-cop-1*, and the mRNA levels of *β’1-COP* in *β’1-cop-2* were around 40% of wild type levels (Figure 2A). These results indicate that *β’1-cop-2* is a knockdown mutant and *β’1-cop-1* is a knockout (KO) mutant. Therefore, for the following experiments, we used *β’1-cop-1*, a *β’1-COP* mutant with only one T-DNA insertion confirmed by Next-Generation Sequencing (NGS).

*β’2-cop-1* (SALK_056771) is the only T-DNA mutant of *β’2-COP* found in the SALK collection (Supplementary Figure S2), and it has mRNA levels of *β’2-COP* around 2% of wild type levels (Figure 2B).

Three *β’3-cop* T-DNA insertion mutants from the SALK collection, *β’3-cop-1* (SALK_004817), *β’3-cop-2* (SALK_206870) and *β’3-cop-3* (SALK_096549), were characterized (Supplementary Figure S2). mRNA levels detected by RT–qPCR of *β’3-cop-1* and *β’3-cop-3* were 18% and 50% of wild type levels, respectively (Figure 2C). No *β’3-COP* mRNA could be detected in *β’3-cop-2* (Figure 2C). When the mRNA levels of *β’3-COP* were also analyzed in *β’3-cop-1* by RT-sqPCR with primers on both sides of the T-DNA insertion (primers RPB’3 and LPB’3), not only a reduction in the expression was detected but also the molecular weight of the band obtained was smaller in the mutant compared to wild type (Supplementary Figure S2B). The sequence of this band suggests that the exon number 23 has been abnormally spliced out, likely due to the T-DNA insertion in the mutant. In *β’3-cop-3*, RT-sqPCR showed that the *β’3-COP* mRNA levels detected by RT–qPCR (50% wild type levels) were due to the presence of truncated transcripts down the T-DNA insertion and showed that this mutant indeed lacked the full length *β’3-COP* transcript (Supplementary Figure S2B, Fragment 2). In summary, the results obtained indicate that *β’3-cop-2* and *β’3-cop-3* are *β’3-COP* knockout mutants. On the other hand, *β’3-cop-1* may contain reduced protein levels of a truncated *β’3-COP* (lacking the last 141 amino acids) that may be partially functional.

None of the *β’2-cop* and *β’3-cop* mutants showed any obvious phenotypic alteration under standard growth conditions when compared to wild type plants, and only *β’1-cop-1* mutant showed slightly reduced plant height (Supplementary Figure S2C).

### 3.2. β’-Cop Double Mutants Showed Different Growth Phenotypes

Next, single mutants were crossed to obtain double mutants. No homozygous *β’1β’2-cop* double mutants were obtained when *β’1-cop-1* and *β’2-cop-1* plants were crossed. In total, we screened 97 F2 plants but failed to recover homozygous *β’1-cop*/*β’1-cop β’2-cop*/*β’2-cop* plants. Furthermore, we did not identify any *β’1-cop*/*β’1-cop β’2-COP*/*β’2-cop* or *β’1-cop*/*β’1-COP β’2-cop*/*β’2-cop* plants either, suggesting that loss of both *β’1-COP* and *β’2-COP* genes compromises viability of the *β’1β’2-cop* double mutant and that *β’3-COP* cannot compensate for it.

**Figure 3:**
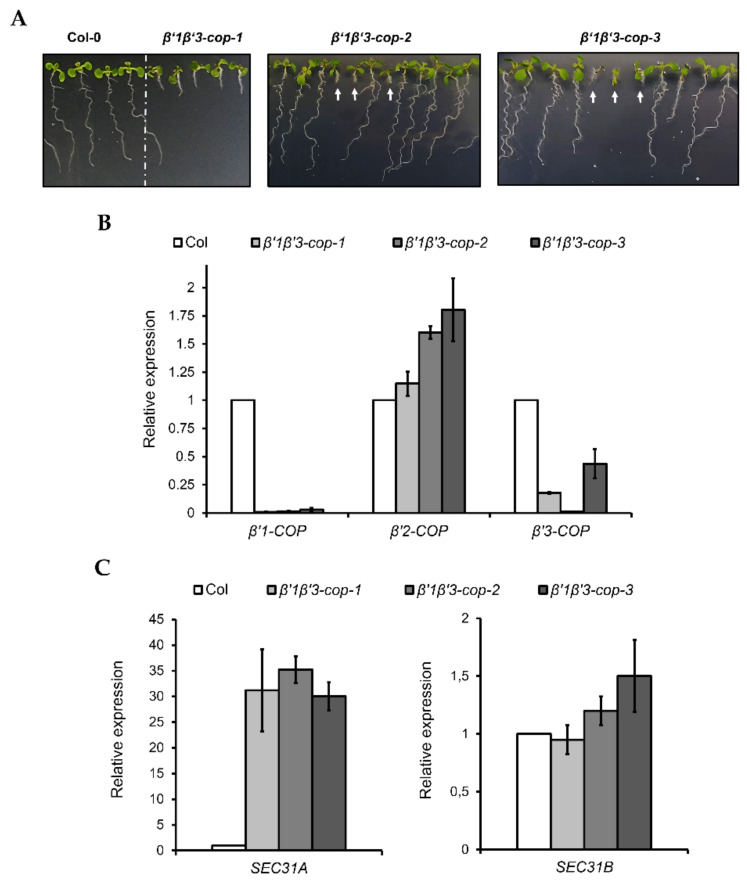
Characterization of *β’1β’3-cop* double mutants. (**A**) All the *β’1β’3-cop* homozygous double mutants show a dwarf phenotype at 7-day-old seedling stage. At later stages, the *β’1β’3-cop-1* mutant also showed a dwarf phenotype with smaller rosette leaves, shorter stems and roots and reduced fertility (Supplementary Figure S3). No homozygous lines could be obtained of *β’1β’3-cop-2* and *β’1β’3-cop-3* mutants as they were only viable as seedlings and failed to develop beyond the seedling stage. White arrows point *β’1β’3-cop-2* and *β’1β’3-cop-3* homozygous seedlings obtained from seeds of *β’1-cop-1*/*β’1-COP β’3-cop-2*/*β’3-cop-2* plants and *β’1-cop-1*/*β’1-cop-1 β’3-COP*/*β’3-cop-3* plants, respectively. (**B**) RT–qPCR analysis show the expression levels of the three *β’-COP* genes in *β’1β’3-cop* double mutants relative to the wild type (Col-0). (**C**) *β’1β’3-cop-1*, *β’1β’3-cop-2* and *β’1β’3-cop*-3 mutants show upregulation of the COPII subunit SEC31A gene. Expression of *SEC31A* and *SEC31B* was analyzed by RT–qPCR. Total RNA was extracted from 7-day-old seedlings of wild type (Col-0) and homozygous mutants (*β’1β’3-cop-2* and *β’1β’3-cop-3* were selected by size). The mRNA was analyzed by RT–qPCR with specific primers and normalized to *UBQ10* expression (Supplementary Tables S1–S3). Results are from three biological samples and three technical replicates. mRNA levels are expressed as relative expression levels and represent fold changes of mutant over wild type. Values represent mean ± s.e.m. of the three biological samples.

*β’1-cop-1* plants were crossed with *β’3-cop-1*, *β’3-cop-2* and *β’3-cop-3* plants to obtain *β’1β’3-cop-1*, *β’1β’3-cop-2* and *β’1β’3-cop-3* double mutants, respectively. The mutant *β’1β’3-cop-1* showed a dwarf phenotype with smaller rosette leaves, shorter stems and roots and reduced fertility (Figure 3 and Figure S3). This double mutant is knockout (KO) for *β’1-COP* but may express reduced protein levels of *β’3-COP*, as described before. However, *β’1β’3cop-2* and *β’1β’3cop-3* mutants that were KO for both *β’1-COP* and *β’3-COP* were only viable as seedlings, albeit strongly reduced in size, and failed to develop beyond the seedling stage (Figure 3A). Interestingly, in these mutants, *β’2-COP* expression was induced 60–80% (Figure 3B), suggesting a possible mechanism of compensation for the loss of *β’1-COP* and *β’3-COP* function. The *β’1β’3-cop-1* mutant likely could develop beyond the seedling stage because it is a knockdown mutant and not a KO mutant for *β’3-COP*. This result indicates that the presence of β’1-COP and β’3-COP is essential for normal seedling development. Therefore, the following experiments were performed with *β’1β’3-cop-1*, as it was not possible to obtain a homozygous line for *β’1β’3cop-2* and *β’1β’3cop-3*.

**Figure 4:**
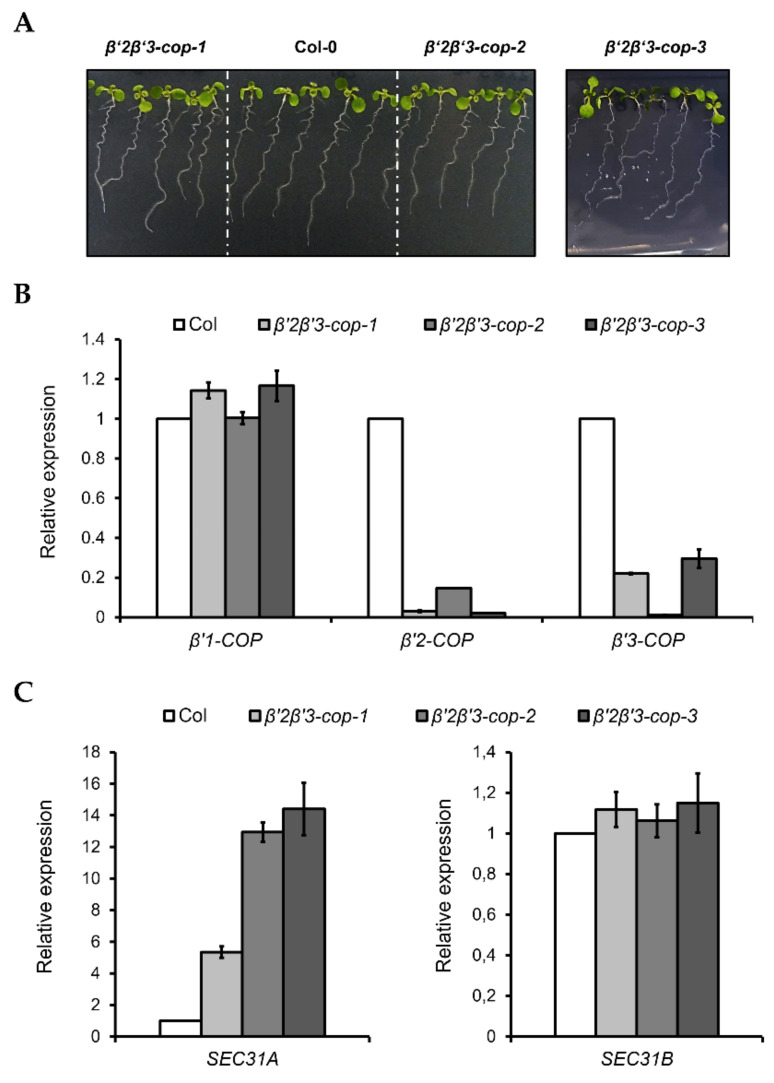
Characterization of *β’2β’3-cop* double mutants. (**A**) All the *β’2β’3-cop* double mutants show a wild type phenotype at 7-day-old seedling stage. (**B**) RT–qPCR analysis show the expression levels of the three *β’-COP* genes. (**C**) *β’2β’3-cop-1*, *β’2β’3-cop-2* and *β’2β’3-cop*-3 mutants show upregulation of the COPII subunit SEC31A gene. Expression of *SEC31A* and *SEC31B* was analyzed by RT–qPCR. Total RNA was extracted from 7-day-old seedlings of the mutants and wild type (Col-0). The mRNA was analyzed by RT–qPCR with specific primers and normalized to *UBQ10* expression (Supplementary Tables S1–S3). Results are from three biological samples and three technical replicates. mRNA levels are expressed as relative expression levels and represent fold changes of mutant over wild type. Values represent mean ± s.e.m. of the three biological samples.

Following the same strategy as before, *β’2-cop-1* plants were crossed with *β’3-cop-1*, *β’3-cop-2* and *β’3-cop-3* plants to obtain *β’2β’3-cop-1*, *β’2β’3-cop-2* and *β’2β’3-cop-3* double mutants, respectively. All the double mutants showed a wild type growth phenotype under standard growth conditions and only showed slightly reduced plant height (Figure 4 and Figure S3). For the following experiments, *β’2β’3-cop-2* (KO for *β’3-COP*) was used. In addition, *β’2β’3-cop-1* (containing *β’3-cop-1*, down for *β’3-COP*) was also used to compare with *β’1β’3-cop-1* (containing *β’3-cop-1*, down for *β’3-COP*), the only homozygous line obtained for the crosses *β’1-cop x β’3-cop*. We have shown previously that depletion of *β-COP* compromises tolerance to NaCl and KCl in *Arabidopsis* [32,33]. Therefore, we decided to test whether *β’2β’3-cop-2*, which has a wild-type phenotype under standard growth conditions, also showed enhanced sensitivity to NaCl and KCl, using the range of NaCl and KCl concentrations used in our previous studies with *β-cop* mutants [32,33]. As it is shown in Supplementary Figure S4, *β’2β’3-cop* mutants had enhanced sensitivity to NaCl and KCl, and this was also the case for *β’1β’3-cop-1*. *β’2β’3-cop-1*, which is a knockdown mutant of *β’3-COP*, showed less sensitivity to NaCl and KCl than *β’2β’3-cop-2* (KO for *β’3-COP*) (Supplementary Figure S4). On the other hand, *β’1β’3-cop-1*, which is also a knockdown mutant of *β’3-COP*, has higher sensitivity than any of the other *β’2β’3-cop* mutants. These results indicate that loss of function of β’-COP, as it happens to loss of function of β-COP, affects tolerance to salt stress which could be due to reduced activity or mislocalization of ions channels/transporters that need COPI for their functional localization [32,33]. Finally, we found that *β’1β’3-cop-1* (Figure 3C), *β’2β’3-cop-1* and *β’2β’3-cop-2* (Figure 4C) showed upregulation of *SEC31A,* that encodes a COPII subunit isoform, but not of *SEC31B*. This specific induction of *SEC31A* was also observed in other mutants affecting COPI function, including α2-COP mutants [30] as well as β-COP mutants [32] and a quadruple mutant affecting p24 family proteins, which are essential for COPI vesicle formation [47].

### 3.3. Loss of Two β’-COP Isoforms Causes a Reduction in the Protein Levels of α-COP

**Figure 5:**
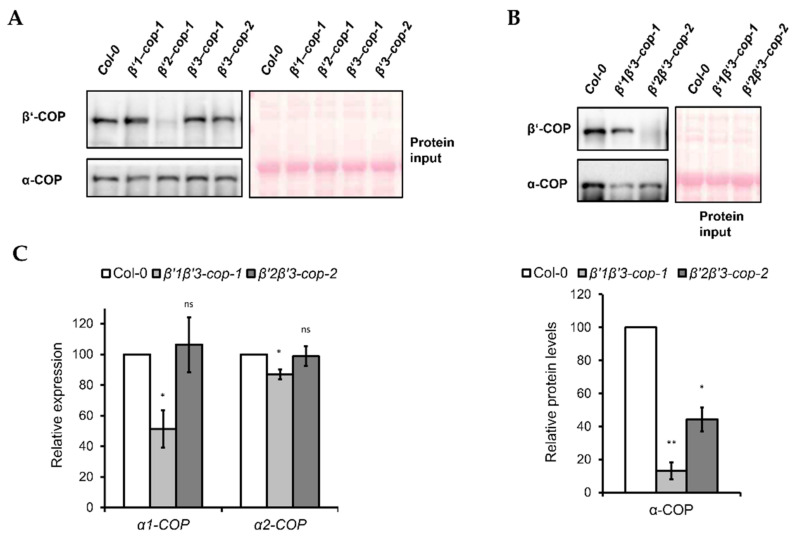
Expression levels of coatomer subunit α-COPI in *β’1β’3-cop*-1 and *β’2β’3-cop-2* mutants. (**A**) Western blot analysis of cytosol protein extracts from cotyledon of 7-day-old seedlings of wild type, *β’1-cop-1*, *β’2-cop-1*, *β’3-cop-1* and *β’3-cop-2* mutants using mammalian β’-COP and α-COP N-terminal peptide antibodies [48,49]. β’1-COP antibodies were raised against the first 12 amino acids of cow β’1-COP. Cow β’-COP and *Arabidopsis* β’1-COP, β’2-COP and β’3-COP share 10, 11 and 10 amino acids, respectively. The β’-COP antibody detected a clear band of approximately 100 kDa, corresponding to the molecular weight of β’-COP, in wild type (Col-0), *β’1-cop-1*, *β’3-cop-1 and β’3-cop-2*, and only a faint band in *β’2-cop-1*, suggesting that mammalian β’-COP antibody has higher affinity for β’2-COP. The different affinity for β’2-COP could be due to the sixth N-terminal amino acid of β’2-COP that is the same in of cow β’-COP and not in β’1-COP and β’3-COP. Alternatively, different splicing forms involved or postranslational modifications at the N-terminal might decrease the affinity of the antibody. α-COP antibodies have been previously shown to recognize both α1-COP and α2-COP isoforms and detected a band of approximately 130 kDa corresponding to the molecular weight of α-COP [30]. (**B**) Western blot analysis of cytosolic protein extracts from cotyledon of 7-day-old seedlings of wild type, *β’1β’3-cop-1* and *β’2β’3-cop-2* using mammalian β’-COP and α-COP N-terminal peptide antibodies. The β’-COP antibody recognized a clear band of approximately 100 kDa in *β’1β’3-cop-1* and a faint band in *β’2β’3-cop-2*, suggesting again that mammalian β’-COP antibody has higher affinity for β’2-COP. Bottom panel shows the relative α-COP protein levels quantified of three biological samples. In (**A**,**B**), 12 μg of total protein was loaded in each lane. Ponceau protein stain was used as a loading control. (**C**) Relative expression levels of *α-COP* genes. Total RNA was isolated from 7-day-old cotyledon seedlings of wild type, *β’1β’3-cop-1* and *β’2β’3-cop-2* mutants. RT-sqPCR analysis was performed with the primers listed in Supplementary Table S2. *ACT7* was used as a control. Values represent mean ± s.e.m. of the three biological samples and were normalized against the band intensity in wild type that was considered to be 100%. Statistical significance: ns, not significant; * *p* < 0.05; ** *p* < 0.01.

Next, we monitored β’-COP protein depletion in the mutants by using an antibody against mammalian β’-COP [48], since there are no *Arabidopsis* β’-COP antibodies available. As shown in Figure 5A, the β’-COP antibody recognized a clear band of approximately 100 kDa, corresponding to the molecular weight of β’-COP, in wild type (Col-0), *β’1-cop-1*, *β’3-cop-1* and *β’3-cop-2* mutants, while in *β’2-cop-1* mutant, only a faint band was detected. The β’-COP antibody also recognized a clear band of approximately 100 kDa in *β’1β’3-cop* and a faint band in *β’2β’3-cop* (Figure 5B). All these results suggest that mammalian β’-COP antibody has a higher affinity for β’2-COP than for the other isoforms.

In yeast, *β’-COP* depletion was shown to affect the levels of other COPI subunits, such as α-COP [11]. Therefore, we tested the effect of *β’-COP* depletion by Western blot analysis using an antibody against mammalian α-COP [49], that has been shown previously to recognize both isoforms of the *Arabidopsis* α-COP subunit [30]. Using this α-COP antibody we found that the levels of α-COP in the single *β’-cop* mutants were not affected (Figure 5A). However, *β’1β’3-cop-1* and *β’2β’3-cop-2* double mutants showed lower levels of α-COP (Figure 5B).

To check whether the decrease in α-COP protein levels found in the *β’1β’3-cop-1* and *β’2β’3-cop-2* double mutants correlated with a decrease in mRNA levels, these were analyzed by RT-sqPCR. As shown in Figure 5C, *β’1β’3-cop-1* (but not *β’2β’3-cop-2*) showed reduced mRNA levels of both *α1-COP* and *α2-COP*, although the reduction in *α1-COP* was much higher than in *α2-COP*.

### 3.4. Loss of Two β’-COP Isoforms Causes Impaired Trafficking of p24δ5, a COPI Dilysine Cargo

**Figure 6:**
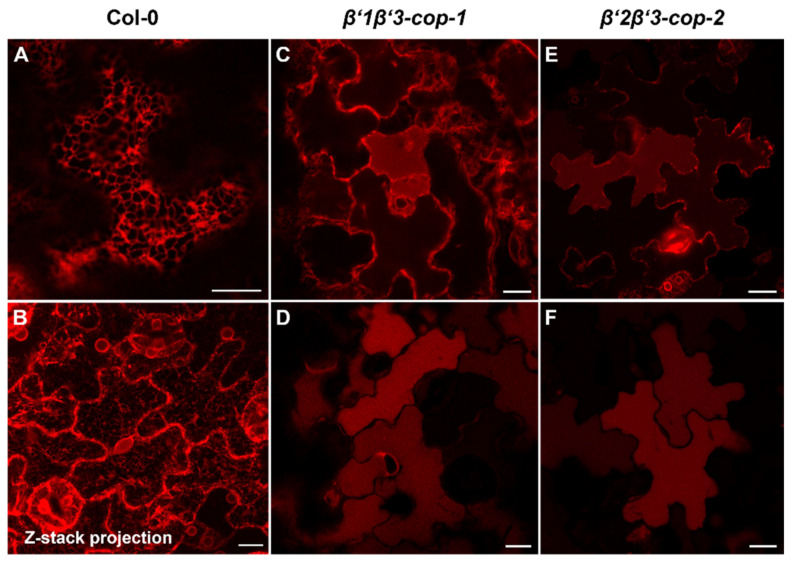
*β’-cop* double mutants show abnormal distribution of RFP–p24δ5, a COPI dilysine cargo. Confocal laser scanning microscopy of epidermal cells of 4.5-day-old cotyledons. All images shown were acquired using comparable photomultiplier gain and offset settings. RFP–p24δ5 mainly localized to the ER network in wild type plants (Col-0) (**A**,**B**) (see a z-stack projection in (**B**)). In contrast, it mainly localized to the vacuole lumen in *β’1β’3-cop-1* (**C**,**D**) and *β’2β’3-cop-2* (**E**,**F**) double mutants, although a partial ER localization was also found (**C**,**E**). Scale bars, 10 μm.

β’-COP has been shown to bind to dilysine motifs, which are present in canonical COPI cargoes. One of these cargoes is p24δ5, a protein of the p24 family, which has been previously shown to localize to the ER due to COPI-dependent Golgi-to-ER transport based on a dilysine motif at its C-terminal tail [36,50]. Therefore, we investigated whether trafficking of RFP–p24δ5 was affected in *β’-COP* double mutants. As shown in Figure 6, RFP–p24δ5 localized to the ER in wild type transgenic plants. In contrast, in both *β’1β’3-cop* and in *β’2β’3-cop* mutants RFP–p24δ5 showed a predominant localization to the vacuole lumen, with some partial ER localization. This is consistent with impaired retrograde trafficking of p24δ5 from the Golgi back to the ER in the mutants and with previous results showing that transport to the vacuole may be a default pathway for membrane proteins in the plant secretory pathway [36]. This result is also consistent with the role of β’-COP in trafficking of dilysine cargoes.

### 3.5. A Double Mutant That Combines New CRISPR/Cas9-Generated β’2-Cop KO Alleles with the β’3-Cop-2 Allele Confirms the β’2β’3-Cop-2 Phenotype

As it has been described above, the mRNA levels of *β’2-COP* in *β’2-cop-1* were around 2% of wild type levels (Figure 2B). On the other hand, the mRNA levels of *β’2-COP* detected in *β’2β’3-cop-2* mutant were around 15% of *β’2-COP* wild type levels (Figure 4B) perhaps due to simultaneous depletion of *β’3-COP*. Therefore, it could not be discarded that the absence of growth phenotype in *β’2β’3-cop-2* was due to remaining *β’2-COP* (15% of wild type). To confirm this, KO *β’2-cop* mutants were generated by CRISPR/Cas9 gene editing. Two sgRNA sequences were designed using ARES-GT software (https://github.com/eugomin/ARES-GT, accessed on 21 December 2021) targeting exons 5 and 7 of *β’2-COP*, respectively (Supplementary Figure S5A). The KO *β’2-cop* mutants were generated in both wild type (Supplementary Figure S5B,C) and *β’3-cop-2* (Supplementary Figure S6) background. As it happened in *β’2-cop-1* (Figure 5A) and *β’2β’3-cop-2* (Figure 5B) mutants, a faint band was detected by Western blot analysis with the mammalian β’-COP antibody in *β’2-cop-cr* (Supplementary Figure S5B) and *β’2β’3-cop-cr* mutants (Supplementary Figure S6A).

The phenotype of *β’2-cop-cr* and *β’2β’3-cop-cr* mutants under standard growth conditions (Supplementary Figure S5C and S6B, respectively) was similar to that of *β’2-cop-1* (Supplementary Figure S2C) and *β’2β’3-cop-2* (Supplementary Figure S3), respectively. These results confirmed that under standard growth conditions, *β’1-COP* can almost completely compensate for the loss of both *β’2-COP* and *β’3-COP*.

Finally, Western blot analysis using α-COP antibody showed that *β’2β’3-cop-cr* double mutants contained lower levels of α-COP, as it happened to the *β’2β’3-cop-2* (Supplementary Figure S6A and Figure 5B, respectively).

## 4. Discussion

Over the last years, several studies have been performed to elucidate putative specific functions of different COPI subunits in mammals. Particularly, a paralog-specific role has been proposed for the γ- and ζ-COP subunits, since these are the only COPI subunits codified by two different genes in mammals [4]. Proteomic studies of COPI vesicles generated in vitro with different γ- and ζ-COP isoforms, using HeLa cells as donor membranes, were not able to reveal a differential protein composition, arguing against selective cargo content [23]. However, it has been recently shown that γ1-COP and γ2-COP isoforms are differentially expressed during the neuronal differentiation of mouse pluripotent cells and, although they are functionally redundant to a large extent, γ1-COP specifically promotes neurite outgrowth [25].

Despite the fact that most COPI genes (including α-, β-, β’-, ε- and ζ-COP) have different paralogs in *Arabidopsis*, it is not yet known whether different COPI subunit isoforms are functionally redundant or may have specific functions, tissue, or development specificity, or perhaps bind different cargo proteins. Interestingly, and in contrast to mammals, morphologically different COPI vesicles have been described in plants [26], which might be formed by different COPI subunit isoforms, although this hypothesis still needs to be demonstrated.

In this work, we have used a loss-of-function approach to analyze the function of the three *Arabidopsis* β’-COP isoforms. To this end, we characterized single and double *β’-COP* mutants. Under standard growth conditions, none of the single *β’-COP* mutants displayed severe developmental defects, which was likely caused by at least partial functional redundancy among *Arabidopsis β’-COP* genes. *β’-COP* double mutant analysis under standard growth conditions suggests that *β’3-COP* cannot compensate for the simultaneous loss of *β’1-COP* and *β’2-COP.* Similarly, *β’2-COP* cannot compensate for the simultaneous loss of *β’1-COP* and *β’3-COP*, as the *β’1β’3-cop* double mutant failed to develop beyond the seedling stage. However, *β’2β’3-cop* double mutants had no major phenotypic alterations, indicating that *β’1-COP* does seem to compensate for the simultaneous lack of *β’2-COP* and *β’3-COP*. The results of double mutant analysis appear to correlate with the seed development expression patterns of *β’1-COP* and suggest a role of *β’1-COP* during seed development that may affect seedling growth.

Coatomer is made of seven equimolar COPI subunits and is recruited *en bloc* from the cytosol onto Golgi membranes. However, it is not clear how the levels of the different COPI subunits are regulated and how the absence of any of the subunits affects the structure of the complex and the stability of the other subunits. In this work, we found that the protein levels of another COPI subunit, α–COP, was not affected in the single *β’-COP* mutants. However, *β’-COP* double mutants showed a dramatic decrease in the levels of α-COP. This is consistent with the known interaction between α- and β’-COP subunits in the B-subcomplex, and suggests that the α–COP subunit is destabilized in the absence of β’-COP. Strikingly, the sec27-1 yeast *β’-COP* mutant (harboring a point mutation in the carboxy-terminal region) also showed a reduction in the levels of α-COP [11]. This was proposed to be due to a local instability of the α-solenoid structure in β’-COP which would affect its interaction with α-COP. Strikingly, *β’1β’3-cop-1*, but not *β’2β’3-cop-2*, have also lower *α1*/*α2-COP* mRNA levels than wild-type, which may also contribute to the decrease in α-COP protein levels in this mutant. As *α1-COP* mRNA levels were more affected than *α2-COP* mRNA levels in *β’1β’3-cop-1*, it would be interesting to test in the future whether the isoform α1-COP is a specific partner of β’1-COP. Further experiments should be performed to clarify these issues.

**Figure 7:**
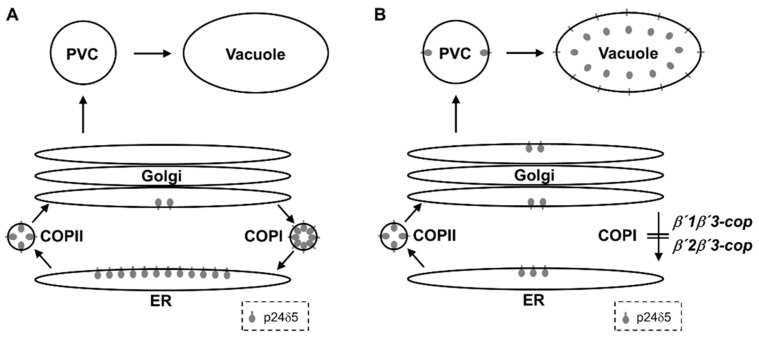
Trafficking of p24δ5 in wild-type and *β’-COP* double mutant plants. (**A**) In wild-type plants, p24δ5 mainly localizes in the ER at steady-state due to efficient, COPI-dependent, Golgi-to-ER transport. (**B**) In *β’-COP* double mutants, p24δ5 is not efficiently retrieved from the Golgi apparatus and thus follows a default pathway to the vacuole, where the luminal part of the protein (including RFP in the case of RFP–p24δ5) is cleaved and released to the vacuole lumen.

The β’-COP subunit has been shown to play a role in binding to dilysine motifs in canonical COPI cargo proteins. Therefore, we hypothesized that loss of β’-COP may affect trafficking of dilysine cargo proteins, as observed in the sec27-1 yeast *β’-COP* mutant [11]. Indeed, we have found that the two *β’1β’3-cop* and *β’2β’3-cop* double mutants showed a mislocalization of p24δ5, which contains a cytosolic C-terminal dilysine motif, from the ER to the vacuole. This may be due to impaired COPI-dependent Golgi-to-ER transport of p24δ5 (Figure 7), which is mediated by its dilysine motif [36]. Indeed, we have shown previously that p24δ5 mutants lacking the dilysine motif were transported along the secretory pathway to the prevacuolar compartment and the vacuole, although a significant fraction was also found at the plasma membrane [36]. This suggests that transport to the vacuole is an alternate default pathway for membrane proteins in the secretory pathway. Therefore, both the absence of the dilysine motif or impaired COPI function have the same trafficking defect in p24δ5, a canonical COPI cargo.

The sequences of *Arabidopsis* β’-COP proteins are very similar. Residues which have been shown to be important for the interaction of β’-COP with dilysine motifs [8] are conserved among the three β’-COP *Arabidopsis* paralogs, and thus one would not expect differential cargo binding among these paralogs. This may explain why *β’1β’3-cop* and *β’2β’3-cop*, although they showed different phenotypes under standard growth conditions, both showed mislocalization of p24δ5. The different phenotypes could be explained by different expression patterns of the β’-COP isoforms, as described above. However, it cannot be discarded that the different β’-COP isoforms have subsets of specific cargoes that could be responsible for the different observed phenotypes.

Altogether, our findings support an essential role of β’1-COP during seedling development. Future experiments should be performed to determine whether this role is due to its tissue or/and development pattern of expression or to a unique function of the β’1-COP isoform.

## Data Availability

The data presented in the current study are available upon request to the corresponding authors.

## Supplementary Materials

The following are available online at https://www.mdpi.com/article/10.3390/cells11060938/s1, Figure S1. Alignment of the protein sequences of *Arabidopsis* β’1-COP, β’2-COP and β’3-COP, Figure S2. Characterization of *β’1-cop*, *β’2-cop* and *β’3-cop* mutants, Figure S3. Adult plant stage phenotype in double mutants, Figure S4. Phenotypic analysis of *β’1β’3-cop-1*, *β’2β’3-cop-1* and *β’2β’3-cop-2* mutants exposed to salt (NaCl) and KCl stress, Figure S5. Characterization of *β’2-cop-cr* mutant, Figure S6. Characterization of *β’2β’3-cop-cr* mutant, Table S1: *β‘1-COP*, *β‘2-COP* and *β‘3-COP* mutants and PCR primers used for their characterization, Table S2. List of primers used for PCR analysis, Table S3. List of primers for RT–qPCR analysis, Table S4. List of CRISPR–Cas9 primers.
