# Supplementary material for: Differential Involvement of Arabidopsis β’-COP Isoforms in Plant Development
Source: Cells. 2022 Mar 9;11(6):938. doi: 10.3390/cells11060938 (PMC8946003; doi:10.3390/cells11060938)
Supplement: Supplementary file 1 [file cells-11-00938-s001.zip › cells-1583693-supplementary.pdf]

cells

MDPI

Differential Involvement of *Arabidopsis* β'-COP Isoforms in Plant Development

Sánchez-Simarro et al., 2022

Supplementary Data

Supplementary Figure S1. Alignment of the protein sequences of *Arabidopsis* β'1-COP, β'2-COP and β'3-COP 2

Supplementary Figure S2. Characterization of *β'1-cop*, *β'2-cop* and *β'3-cop* mutants 3

Supplementary Figure S3. Adult plant stage phenotype in double mutants 4

Supplementary Figure S4. Phenotypic analysis of *β'1β'3-cop-1*, *β'2β'3-cop-1* and *β'2β'3-cop-2* mutants exposed to salt (NaCl) and KCl stress 5

Supplementary Figure S5. Characterization of *β'2-cop-cr* mutant 6

Supplementary Figure S6. Characterization of *β'2β'3-cop-cr* mutant 7

Supplementary Table S1. *β'1-COP*, *β'2-COP* and *β'3-COP* mutants and PCR primers used for their characterization 8

Supplementary Table S2. List of primers used for PCR analysis 9

Supplementary Table S3. List of primers for RT-qPCR analysis 10

Supplementary Table S4. List of CRISPR-Cas9 primers 11

```
1´      MPLRLEIKRKLAQRSERVKSVDLHPTEPWILASLYSGTLCIWNYQTQVMAKSFEVTELPV        60
2´      MPLRLDIKRKFAQRSERVKSVDLHPTEPWILASLYSGTVCIWNYQTQTITKSFEVTELPV        60
3´      MPLRLEIKRKFAQRSERVKSVDLHPTEPWILASLYSGTLCIWNYQTQTMVKSFDVTELPV        60
        *****:****:***************************:********.:.***:******

1´      RSAKFVARKQWVVAGADDMYIRVYNYNTMDKVKVFEAHSDYIRCVAVHPTLPYVLSSSDD        120
2´      RSAKFIPRKQWVVAGADDMYIRVYNYNTMDKVKVFEAHSDYIRCVAVHPTLPYVLSSSDD        120
3´      RSAKFIARKQWVVAGADDMFIRVYNYNTMDKIKVFEAHADYIRCVAVHPTLPYVLSSSDD        120
        *****: ************:***********:******:*********************

1´      MLIKLWDWEKGWACTQIFEGHSHYVMQVTFNPKDTNTFASASLDRTIKIWNLGSPDPNFT        180
2´      MLIKLWDWENGWACTQIFEGHSHYVMQVVFNPKDTNTFASASLDRTIKIWNLGSPDPNFT        180
3´      MLIKLWDWEKGWLCTQIFEGHSHYVMQVTFNPKDTNTFASASLDRTIKIWNLGSPDPNFT        180
        *********:** ***************.*******************************

1´      LDAHQKGVNCVDYFTGGDKPYLITGSDDHTAKVWDYQTKSCVQTLEGHTHNVSAVCFHPE        240
2´      LDAHQKGVNCVDYFTGGDKPYLITGSDDHTAKVWDYQTKSCVQTLDGHTHNVSAVCFHPE        240
3´      LDAHLKGVNCVDYFTGGDKPYLITGSDDHTAKVWDYQTKSCVQTLEGHTHNVSAVSFHPE        240
        **** ****************************************:*********.****

1´      LPIIITGSEDGTVRIWHATTYRLENTLNYGLERVWAIGYIKSSRRVVIGYDEGTIMVKLG        300
2´      LPIIITGSEDGTVRIWHATTYRLENTLNYGLERVWAIGYIKSSRRVVIGYDEGTIMVKLG        300
3´      LPIIITGSEDGTVRIWHATTYRLENTLNYGLERVWAIGHIKGSRRVVIGYDEGSIMVKLG        300
        **************************************:**.***********:******

1´      REIPVASMDNTGKIIWAKHNEIQTANIKSIGADYEVTDGE-------RLPLSVKELGTC-        352
2´      REIPVASMDSSGKIIWAKHNEIQTANIKSIGAGYELSSLYLRLLMEKDFPCLLKSWGPVI        360
3´      REIPVASMDNSGKIIWAKHNEIHTVNIKSVGADEVTD--------GERLPLAVKELGTC-        351
        *********.:***********:*.****:**.               :*  :*. *

1´      ----DLYPQSLKHNPNGRFVVVCGDGEYIIYTALAWRNRSFGSGLEFVWSSEGECAVRES        408
2´      FIHNYSNSQSLKHNPNGRFVVVCGDGEYIIYTALAWRNRSFGSGLEFVWSSEGECAVRES        420
3´      ----DLYPQSLKHNPNGRFVVVCGDGEYIIYTALAWRNRSFGSALEFVWSSDGEHAVRES        407
                ***********************************.*******:** *****

1´      SSKIKIFSKNFQEKRSIRPTFSAEKIFGGTLLAMCSSDFICFYDWAECRLIQRIDVTVKN        468
2´      SSKIKIFSKNFQERKSIRPTFSAEKIFGGTLLAMCSNDFICFYDWAECRLIQQIDVTVKN        480
3´      STKIKIFSKNFQEKKTVRPTFSAEHIFGGTLLTMCSSDFICFYDWAECRLIRRIDVTVKN        467
        *:***********::::*******:*******:***.**************::*******

1´      LYWADSGDLVAIASDTSFYILKFNRDLVTSHFDSGRPTEEEGVEDAFEVLHENDERVRTG        528
2´      LYWAESGDLVAIASDTSFYILKYNRELVSSHFDSGRPTDEEGVEDAFEVLHENDERVRTG        540
3´      LYWADSGDLVAIASDTSFYILKFNRDIVSSYFDGGKQIDEEGIEDAFELLNETNERVRTG        527
        ****:*****************:**::*:*:**.*:  :***:*****:*:*.:******

1´      IWVGDCFIYNNSSWKLNYCVGGEVTTMYHLDRPMYLLGYLASQSRVFLVDKEFNVIGYTL        588
2´      IWVGDCFIYNNSSWKLNYCVGGEVTTMYHLDRPMYLLGYIANQSRVYLVDKEFNVIGYTL        600
3´      LWVGDCFIYTNSSWRLNYCVGGEVTTMYHLDRPMYLLGYLANQSRVYLIDKEFNVIGYTL        587
        :********.****:************************:*.****:*:***********

1´      LLSLIEYKTLVMRGDLDKASEILPTIPKDQHNSVAHFLESRGMIEDALEIATDPDYRFEL        648
2´      LLSLIEYKTLVMRGDLDRANQILPTIPKEQHNNVAHFLESRGMIEDALEIATDPDYKFDL        660
3´      LLSLIEYKTLVMRGDLEQANEVLPSIPKEHHNSVAHFLESRGMTEDALEVATDPDYRFEL        647
        ****************::*.::**:***::**.********** *****:******:*:*

1´      AIQLGRLEIAQEIAVEVQSESKWKQLGELAMSSGKLQMAEECMKYAMDLSGLLLLYSSLG        708
2´      AIQLGRLEIAKEIAEEVQSESKWKQLGELAMSSGKLQLAEDCMKYAMDLSGLLLLYSSLG        720
3´      AIQLGRLAVAKDIAVEAQNESKWKQLGELAMSSGKLDMAEECMRHAMDLSGLLLLYSSLG        707
        ******* :*::** *.*.*****************::**:**::***************

1´      DAEGVTKLATLAKEQGKNNVAFLCLFMLGKLEDCLQLLVESNRIPEAALMARSYLPSKVS        768
2´      DAEGVSKLACLAKEQGKNNVAFLCLFTLGRLEDCLQLLVESNRIPEAALMARSYLPSKVS        780
3´      DADGMMKLAALAKEQGKNNVAFLCLFMLGQVEDCLHLLVESNRIPEAALMARSYLPSKVS        767
        **:*: *** **************** **::****:************************

1´      EIVALWRKDLSKVNSKAAESLADPEEYSNLFEDWQVALSVEAKAVETRGVYTGAKDYPSH        828
2´      EIVALWREDLSKVNPKAAESLADPEEYSNLFEDWQVALSVEANTAETRGVYTAAENYPSH        840
3´      EIVALWRNDLTKISPKAAESLADPEEYPNLFEEWQVALSLENRAAETRGVHPPAGDYCSH        827
        *******:**:*:. ************ ****:******:* .:.*****:  * :* **

1´      ADKSSMTLVEAFRNLQVEEEESLENGDMDHEEVVAEENGNEQRNEDDVAEHVEEHHEEKE        888
2´      ADKPSITLVEAFRNLQVEAEESLENGNIDHEVA--EENGHVENEG------DEEEQQEEE        892
3´      ADRDHTTLVDAFRIMQIEEEGRLEQGDVLDEVGEEGEDGEEEEEEDRQEES--SDGRQQN        885
        **:   ***:*** :*:* *  **:*:: .*     *:*. :.:         .. .:::

1´      AEEEEGIVDGDSTDGAVLVNGSEADEEWGTNNEGNPSA  926
2´      VNEEEGVVDADSTDGAVLVNGSEGEEEWGTNNKGNPSA  930
3´      VEEEAVVVDADSTDGAVLVNGNESEEQWVLTPPQE---  920
        .:**  :**.***********.*.:*:*  .   :
```

Supplementary Figure S1. Alignment of the protein sequences of *Arabidopsis* β′1-COP, β′2-COP and β′3-COP. ClustalW software was used to generate the alignment.

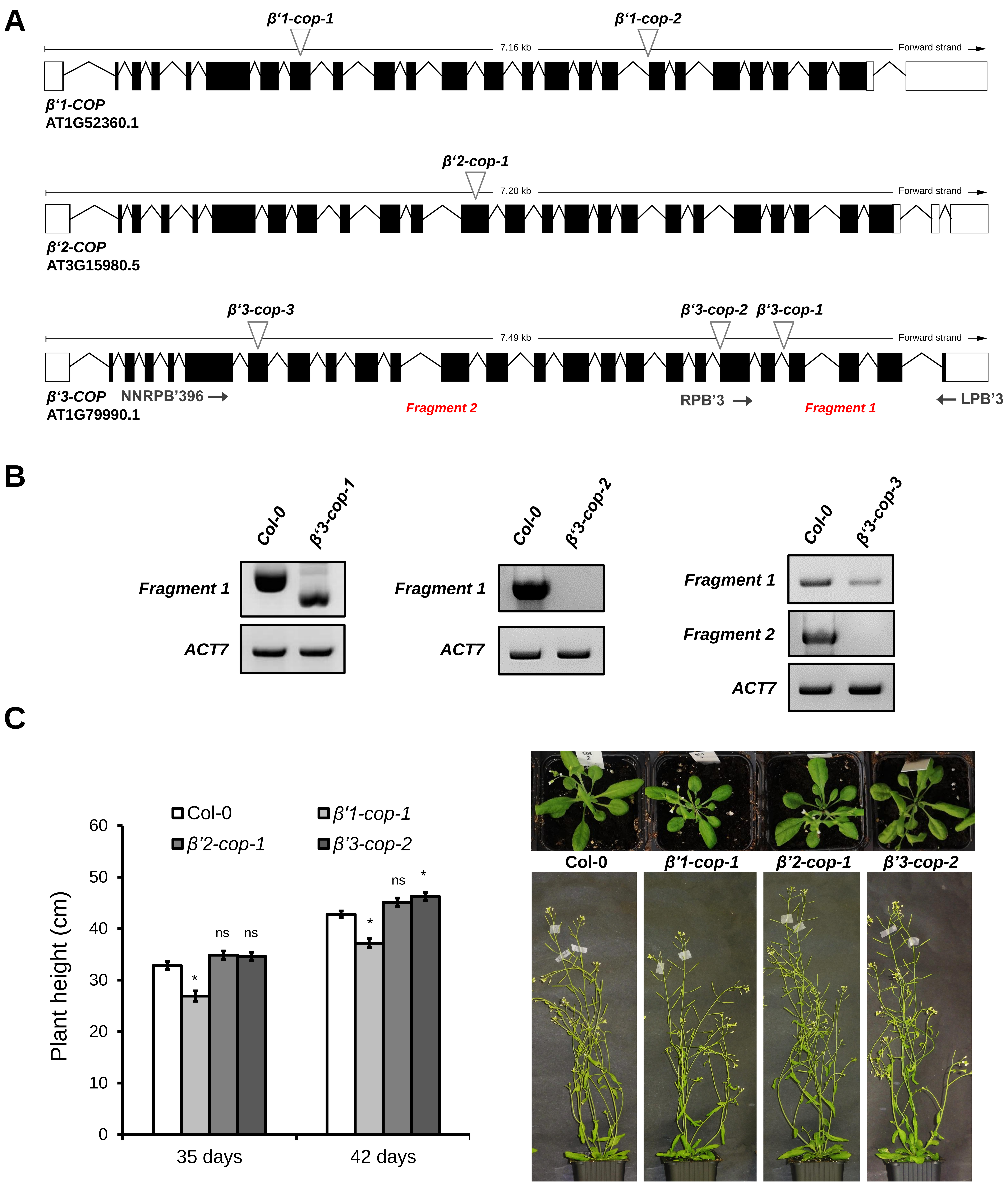

Supplementary Figure S2. Characterization of *β’1-cop*, *β’2-cop* and *β’3-cop* mutants. A. Diagram of *β’1-COP, β’2-COP* and *β’3-COP* genes and localization of the T-DNA insertion (triangle) in the different mutants described in the text. Black boxes represent coding regions. **B.** RT-sqPCR analysis was done to detect truncated transcripts downstream the T-DNA insertion (Fragment 1) in *β’3-cop-2* and *β’3-cop-3* mutants and the full *β’3-COP* transcript in *β’3-cop-1* mutant (Fragment 1) and in *β’3-cop-3* mutant (Fragment 2). The position of the primers used are shown by arrows in panel A and described in Supplementary Table S2: RPB´3 and LPB´3 (Fragment 1) and NNRPB´396 and LPB´3 (Fragment 2). Total RNA from 7-day-old seedlings of the mutants and wild type (Col-0) were used for the RT-sqPCR. *ACT7* was used as a control. **C.** Right, adult phenotype of the mutants and Col-0 at 35 days of growth. Left, the height of 35- and 42-day-old plants expressed as mean±s.e.m. (n=4). Statistical significance: ns, not significant; *p < 0.05.

A

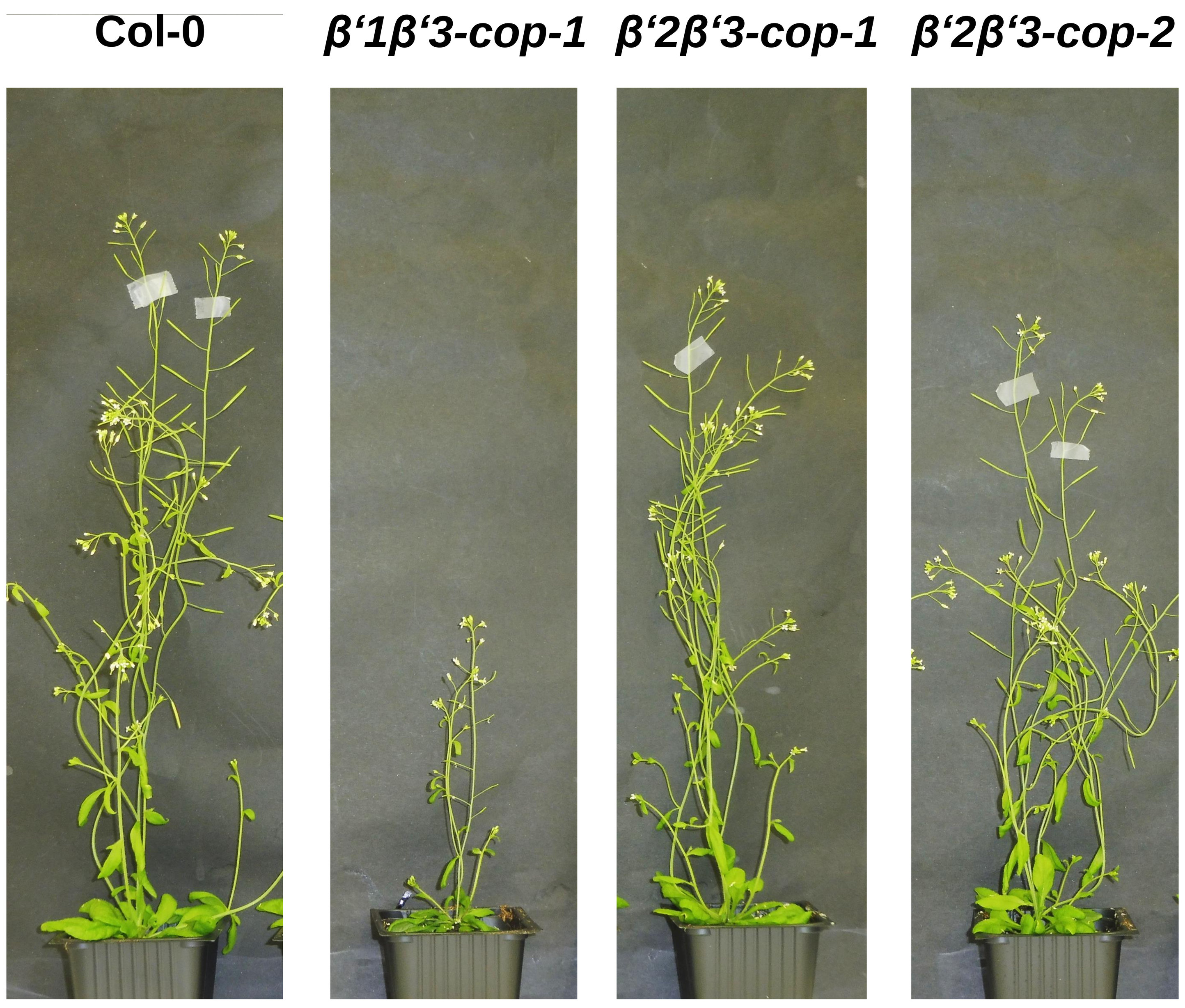

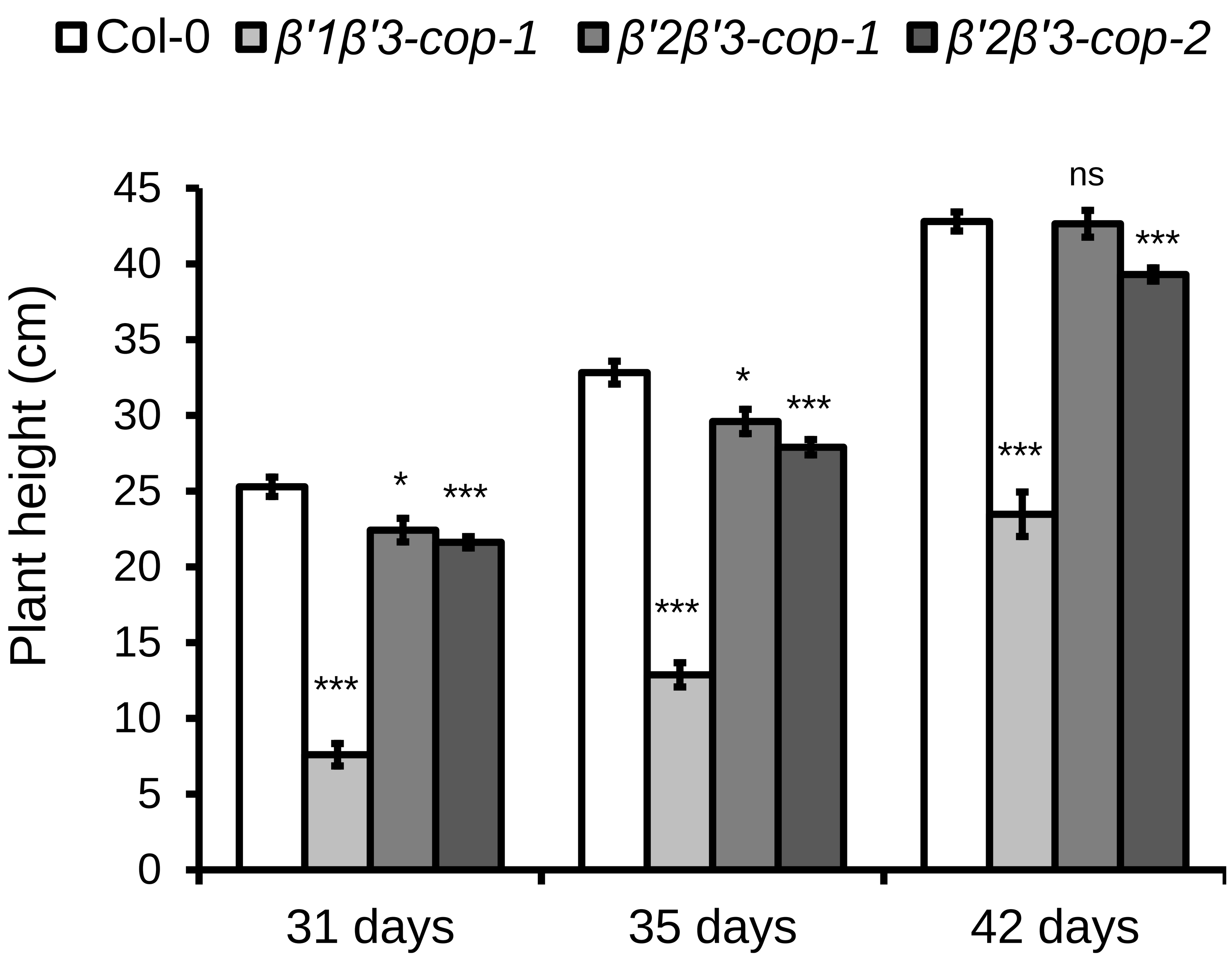

B

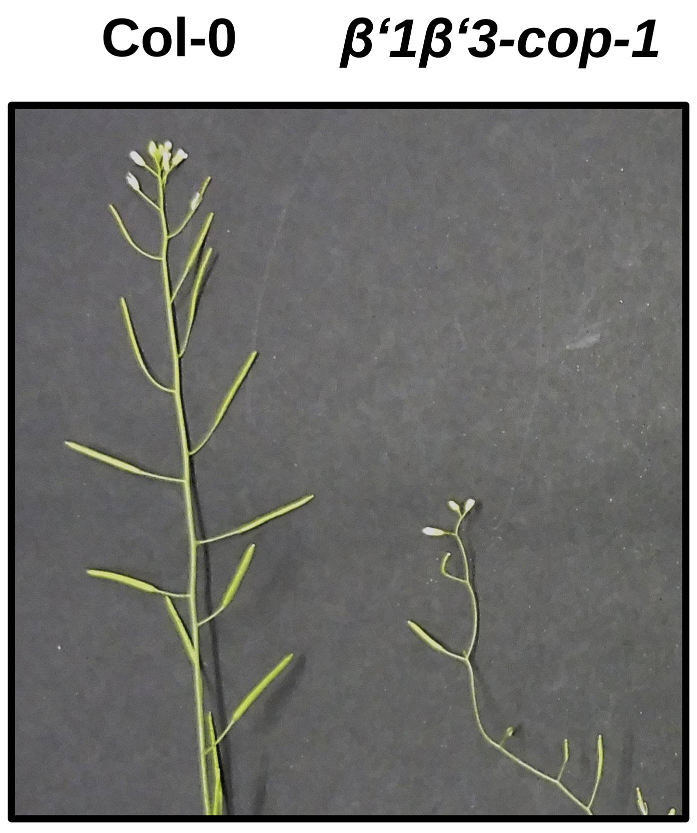

Supplementary Figure S3. Adult plant stage phenotype in double mutants. A. Left panel shows a dwarf phenotype in *β′1β′3-cop-1*. Right panel shows plant height measured at 31, 35 and 42 days of growth in *β′1β′3-cop-1, β′2β′3-cop-1* and *β′2β′3-cop-2* mutants and expressed as mean±s.e.m. (n=4). **B.** Wild type and *β′1β′3-cop-1* inflorescences. Statistical significance: ns, not significant; *$p < 0.05$; ***$p < 0.001$.

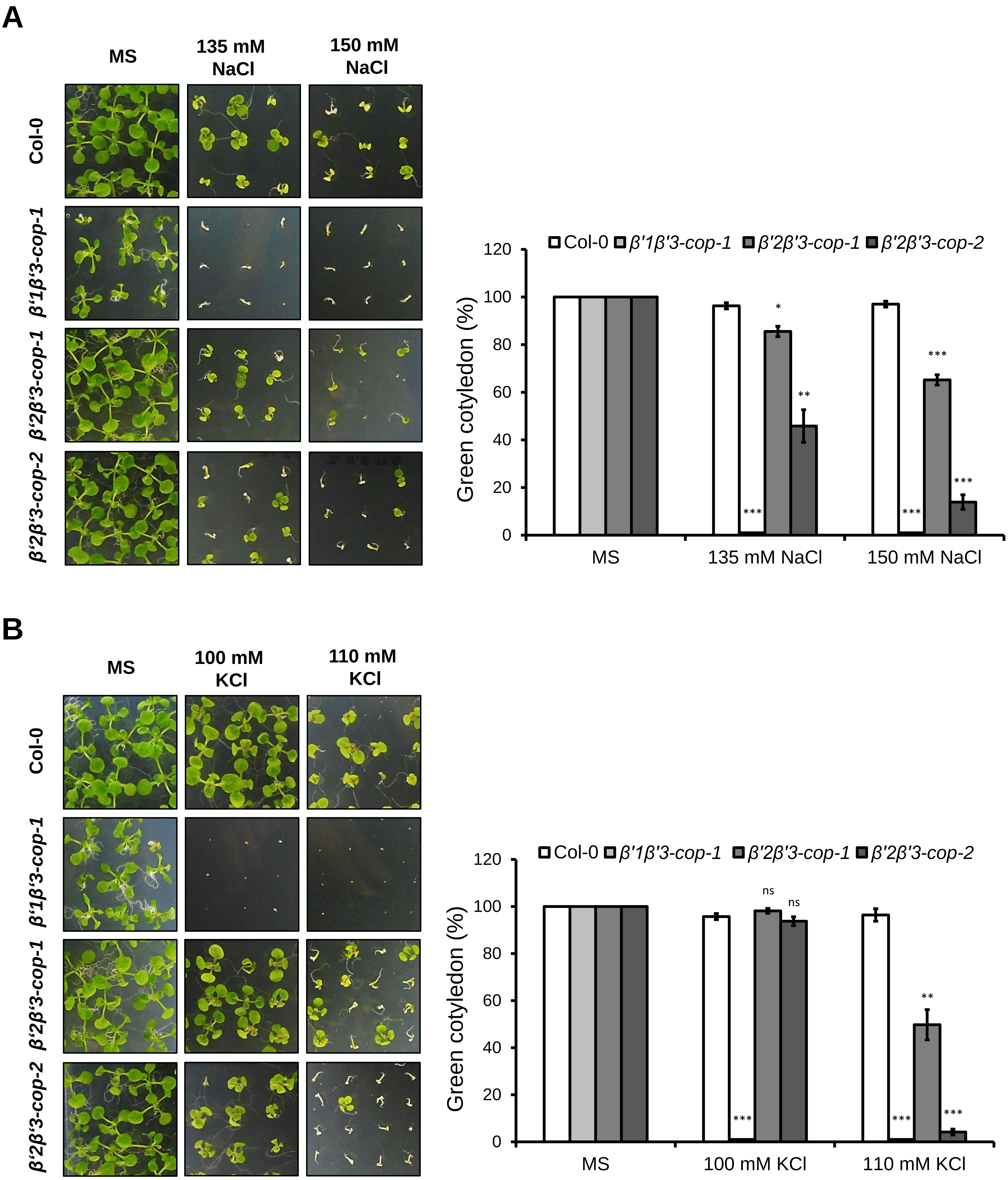

Supplementary Figure S4. Phenotypic analysis of *β'1β'3-cop-1*, *β'2β'3-cop-1* and *β'2β'3-cop-2* mutants exposed to salt (NaCl) and KCl stress. Wild type (Col-0), *β'1β'3-cop-1*, *β'2β'3-cop-1* and *β'2β'3-cop-2* seeds were sown on 0.5× MS for control conditions and 0.5× MS supplemented with 135 mM NaCl and 150 mM NaCl (**A**) or supplemented with 100 mM KCl and 110 mM KCl (**B**) in Petri plates. The right panels show the percentage of seedlings with green cotyledons, which was calculated after 12 days. Data are mean ± s.e.m. (n=144) of four independent experiments. Statistical significance: ns, not significant; *$p < 0.05$; **$p < 0.01$; ***$p < 0.001$.

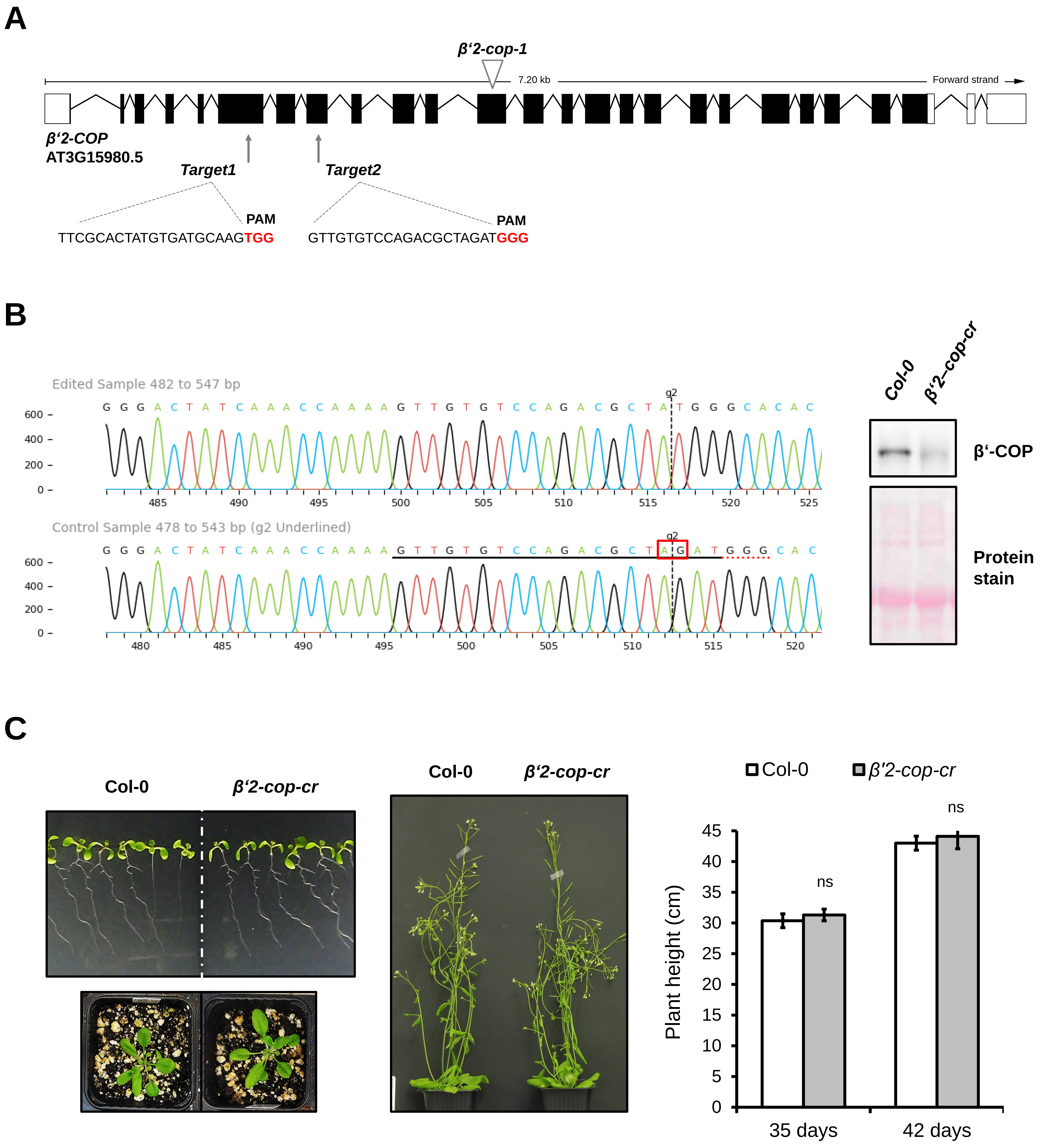

Supplementary Figure S5. Characterization of *β′2-cop-cr* mutant. A. Diagram of *β′2-COP* gene and localization of the T-DNA insertion (triangle) in the *β′2-cop-1* mutant and the two specific target regions (Target1 and Target2) for CRISPR-Cas9 machinery. PAM sequence is showed in red. Black boxes represent coding regions. **B.** CRISPR-Cas9 mutation in the second gRNA target region. Chromatogram represents the sequence of the *β′2-COP* gene modified by CRISPR-Cas9 (upper chromatogram), and the corresponding region in Col-0 (lower chromatogram). The black line, dashed-red line and red box indicate the gRNA target region, the PAM sequence and the deleted two nucleotides, respectively. The right panel shows the Western blot analysis done with cytosol protein extracts from 7-day-old cotyledon wild type and *β′2-cop-cr* mutant seedlings using the cow β′-COP antibody. 10 µg of total protein was loaded in each lane. Ponceau protein stain was used as a loading control. **C.** *β′2-cop-cr* mutant shows a wild type phenotype at each stage of development (7-, 21- and 35-day-old plants). Right panel, the height of 35- and 42-day-old plants expressed as mean±s.e.m. (n=5). Statistical significance: ns, not significant.

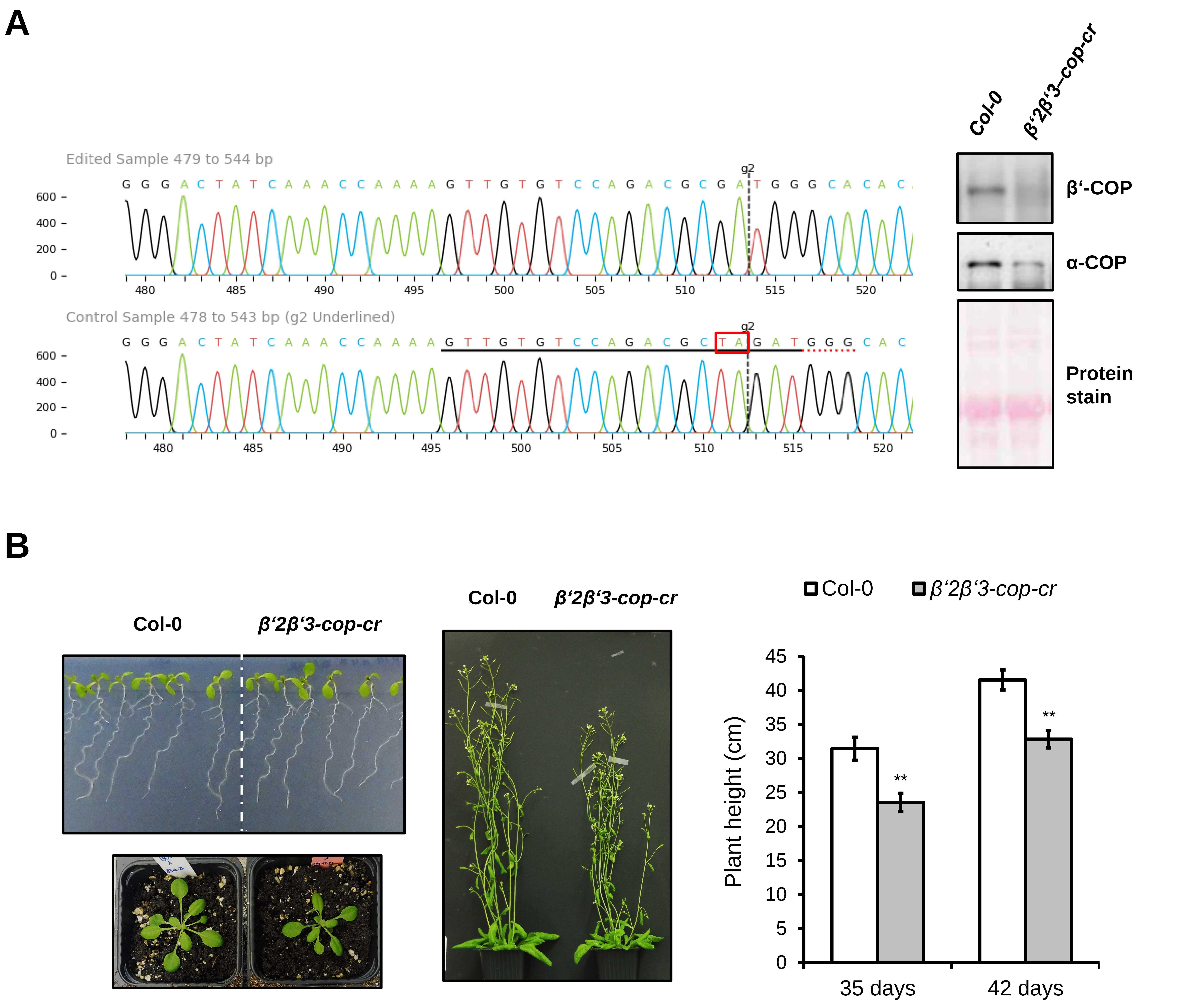

Supplementary Figure S6. Characterization of *β′2β′3-cop-cr* mutant. A. CRISPR-Cas9 mutation in the second gRNA target region. Chromatogram represents the sequence of the *β′2-COP* gene modified by CRISPR-Cas9 (upper chromatogram), and the corresponding region in Col-0 (lower chromatogram). The black line, dashed-red line and red box indicate the gRNA target region, the PAM sequence and the deleted two nucleotides, respectively. The right panel shows Western blot analysis done with cytosol protein extracts from 7-day-old cotyledon wild type (Col-0) and *β′2β′3-cop-cr* mutant seedlings using the cow β′-COP /α-COP antibodies. 10 μg of total protein was loaded in each lane. Ponceau protein stain was used as a loading control. **B.** *β′2β′3-cop-cr* mutant and wild type showed similar phenotypes and only a mild reduction in plant height at adult stage of development is detected in the *β′2β′3-cop-cr* mutant. Left panel show the phenotype of 7-, 21- and 35-day-old plants. Right panel, the height of 35- and 42-day-old plants expressed as mean±s.e.m. (n=5). Statistical significance: **$p < 0.01$.

Supplementary Table S1. ***β'1-COP, β'2-COP*** **and** ***β'3-COP*** **mutants and PCR primers used for their characterization.**

Gene	Mutant	Genotyping primers		RT-PCR primers	RT-qPCR primers
		T-DNA insertion	Wild type allele		
β'1-COP At1g52360	*β'1-cop-1* (SALK_206753)	LBb1/RPB'1	LPB'1/RPB'1	-	qB1primaR qB1primaF2
	β'1-cop-2 (WiscDsLoxHs036_02G)	L4/RPB'136	LPB'136/RPB'136	-	qB1primaR qB1primaF2
β'2-COP At3g15980	*β'2-cop-1* (SALK_056771)	LBb1/RPB'2	LPB'2/RPB'2	-	qB2primaR qB2primaF
β'3-COP At1g79990	*β'3-cop-1* (SALK_004817)	LBb1/RPB'3	LPB'3/RPB'3	LPB'3/RPB'3	qB3primaR qB3primaF1
	β'3-cop-2 (SALK_206870)	LBb1/LPB'3	LPB'3/NLPB'3	LPB'3/RPB'3	qB3primaR qB3primaF1
	β'3-cop-3 (SALK_096549)	LBb1/RPB'396	NNLPB'3/NNRPB'396	LPB'3/RPB'3 LPB'3/NNRPB'396	qB3primaR qB3primaF1

Supplementary Table S2. List of primers used for PCR analysis

β' primers	Gene	Sequence (5' → 3')
RPβ1'	*β'1-COP* At1g52360	TGTAACCAATTGCCCAGACTC
LPβ1'	*β'1-COP* At1g52360	CGGCTTTTGTTCTTTTGTGTC
LPB'136	*β'1-COP* At1g52360	CTCTCCACCAATAACTGCAA
RPB'136	*β'1-COP* At1g52360	GAGTCTGGGCAATTGGTTACA
RPβ2'	*β'2-COP* At3g15980	TGTATTCTGGCATGGGAAAAC
LPβ2'	*β'2-COP* At3g15980	CCAACTCTCGCTGTAATAGCG
RPβ3'	*β'3-COP* At1g79990	ATGCAGATGGAATGATGAAGC
LPβ3'	*β'3-COP* At1g79990	CTTCACTCTTCACCAACCCTA
NNLPB'3	*β'3-COP* At1g79990	GTCTTCACTCTTCACCAACCCTACTCTTGT
NNRPB'3 96	*β'3-COP* At1g79990	CTCATGCTGATTACATTCGCTGTGTTGC
NLPB'3 (12)	*β'3-COP* At1g79990	AGTAGCAACTGATCCTGACTACA
**LB primers**		
LBb1	T-DNA	GGATCCGCGTGGACCGCTTGCTGCAACT
L4	T-DNA	TGATCCATGTAGATTTCCCGGACATGAAG
**Housekeeping primers**		
A5	*ACT7* At5g09810	GGATCCAAATGGCCGATGGTGAGG
A3	*ACT7* At5g09810	GGAAAACTCACCACCACGAACCAG
**COPI related primers**		
LPalfa1	*α1-COP* At1g62020	AGAATTACCTTGGCGAAGAGC
NRPalfa1	*α1-COP* At1g62020	GGATCCGTGCCATTATCGTTGAGAGATT
LPNGalfa2	*α2-COP* At2g21390	GGATCCCATAATCATTCTGACTTGT
RPGalfa2	*α2-COP* At2g21390	GCGTACCAGCAGACAAAGAAC

Supplementary Table S3. List of primers for RT-qPCR analysis

β' primers	Gene	Sequence (5' → 3')
qB1primaR	*β'1-COP* At1g52360	ACATTGGCCTTGACCGACTTGCT
qB1primaF2	*β'1-COP* At1g52360	GAGGAGCATCATGAAGAGAAAGAAGC
qB2primaR	*β'2-COP* At3g15980	CCTGTTGTTCCTCTTCATCTCCTTC
qB2primaF	*β'2-COP* At3g15980	ACCGCTGCAGAAAATTATCCATCCCA
qB3primaR	*β'3-COP* At1g79990	GTCTTCACTCTTCACCAACCCTACTC
qB3primaF1	*β'3-COP* At1g79990	GGGATGTTCTCGACGAGGTTG
**Housekeeping primers**		
UBQ10F	*UBQ10* At4g05320	GGCCTTGTATAATCCCTGATGAATAAG
UBQ10R	*UBQ10* At4g05320	AAAGAGATAACAGGAACGGAAACATAGT
**COPI related primers**		
Sec31AF	*SEC31A* At1g18830	AACGTGATTTTGGTGCAGCGTTA
Sec31AR (Sec31AIN3)	*SEC31A* At1g18830	TGGAAGCCAAGAACTGCACTCATC
Sec31BF	*SEC31B* At3g63460	CAGCAGCTGGACCCATAGGATTTAC
Sec31BR	*SEC31B* At3g63460	GCTGTGTTGGAGGACTTGCTGGTTG

Supplementary Table S4. List of CRISPR-Cas9 primers

Primers	Gene	Sequence (5' → 3')
gbB2-316F	gRNA-1<br>*β'2-COP*<br>At3g15980	GTGCATTCGCACTATGTGATGCAAG
gbB2-316R	gRNA-1<br>*β'2-COP*<br>At3g15980	AAACCTTGCATCACATAGTGCGAAT
gbB2-349F	gRNA-2<br>*β'2-COP*<br>At3g15980	GTGCAGTTGTGTCCAGACGCTAGAT
gbB2-349R	gRNA-2<br>*β'2-COP*<br>At3g15980	AAACATCTAGCGTCTGGACACAACT
GBS1	pUPD2 vector<br>CRISPR-Cas9	GCTTTCGCTAAGGATGATTTCTGG
GBS2	pUPD2 vector<br>CRISPR-Cas9	CAGGGTGGTGACACCTTGCC
C31	*β'2-COP*<br>At3g15980<br>PCR	GCCTTGTATAGTCTGTCGCTGTTA
C33	*β'2-COP*<br>At3g15980<br>PCR	TCGTTGTTGATGGTCCCGTA
C32	*β'2-COP*<br>At3g15980<br>Sequencing	TCCGACCCTTCCATATGTGC
